# A protocol to identify non-classical risk factors for preterm births: the Brazilian Ribeirão Preto and São Luís prenatal cohort (BRISA)

**DOI:** 10.1186/1742-4755-11-79

**Published:** 2014-11-19

**Authors:** Antônio Augusto Moura da Silva, Vanda Maria Ferreira Simões, Marco Antonio Barbieri, Viviane Cunha Cardoso, Claudia Maria Coelho Alves, Erika Bárbara Abreu Fonseca Thomaz, Rejane Christine de Sousa Queiroz, Ricardo Carvalho Cavalli, Rosângela Fernandes Lucena Batista, Heloísa Bettiol

**Affiliations:** Departamento de Saúde Pública, Universidade Federal do Maranhão (UFMA), Rua Barão de Itapary, 155 Centro, 65020-070 São Luís, Maranhão Brazil; Faculdade de Medicina de Ribeirão Preto, Universidade de São Paulo, Ribeirão Preto, São Paulo, Brazil

**Keywords:** Cohort studies, Prenatal care, Premature birth, Risk factors, Stress, psychological, Infection

## Abstract

**Background:**

Preterm birth is the main cause of morbidity and mortality during the perinatal period. Classical risk factors are held responsible for only 1/3 of preterm births and no current intervention has produced an appreciable reduction of this event. It is necessary to explore new hypotheses and mechanisms of causality by using an integrated approach, collaboration among research groups and less fragmented theoretical-methodological approaches in order to detect new risk factors and to formulate more effective intervention strategies.

**Methods:**

The study will be conducted on a convenience cohort of Brazilian pregnant women recruited at public and private prenatal health services. A total of 1500 pregnant women in São Luís, and 1500 in Ribeirão Preto, will be invited for an interview and for the collection of biological specimens from the 22nd to the 25th week of gestational age (GA). At the time of delivery they will be reinterviewed. GA will be determined using an algorithm based on two criteria: date of last menstruation (DLM) and obstetric ultrasound (OUS) performed at less than 20 weeks of GA. Illicit drug consumption during pregnancy will be determined using a self-applied questionnaire and the following instruments will be used: perceived stress scale, Beck anxiety scale, screening for depression of the Center of Epidemiological Studies (CES-D), experiences of racial discrimination, social network and social support scale of the Medical Outcomes Study and violence (Abuse Assessment Screening and violence questionnaire of the WHO). Bacterial vaginosis, urinary tract infection and periodontal disease will also be identified. Neuroendocrine, immunoinflammatory and medical intervention hypotheses will be tested. The occurrence of elective cesarean section in the absence of labor will be used as a marker of medical intervention.

**Conclusion:**

Psychosocial, genetic and infectious mechanisms will be selected, since there are indications that they influence preterm birth (PTB). The studies will be conducted in two Brazilian cities with discrepant socioeconomic conditions. The expectation is to identify risk factors for PTB having a greater predictive power than classically studied factors. The final objective is to propose more effective interventions for the reduction of PTB, which, after being tested, might subsidize health policies.

**Electronic supplementary material:**

The online version of this article (doi:10.1186/1742-4755-11-79) contains supplementary material, which is available to authorized users.

## Background

Preterm birth (PTB) is currently one of the most important perinatal problems because of its association with significant morbidity and mortality at the beginning of life [1–3]. Its prevalence is high and is increasing in developed countries [4, 5] and in some Brazilian cities [6, 7].

The etiology of PTB is not well known [8]. Gestational risk factors such as infections, multiple births, hypertension, smoking habit and the use of illicit drugs, exhausting work, low body mass index, insufficient weight gain, low maternal schooling, black race, and a history of preterm births have been demonstrated to be responsible for only one third of preterm deliveries [9, 10]. These conditions seem to be markers of more distal causes that need to be identified [11]. Thus, other etiologies should be better studied.

There is growing evidence that maternal prenatal stress is a potent risk factor for adverse birth outcomes, an effect that may be mediated by biological and behavioral factors [12, 13]. Ruiz et al. [14] reported that disequilibrium of maternal hormonal homeostasis caused by stress might contribute to a significant proportion of preterm births.

The racial question has also received attention regarding the etiology of PTB, with black women presenting a two to three times higher chance of delivering preterm than white women [15]. Several studies have demonstrated an association between the experience of racial discrimination and an increased risk of PTB and low birth weight [16–18].

Another situation that is being related to worse neonatal outcomes is violence during pregnancy. A study conducted in India detected a 2.59 times higher risk of perinatal and neonatal mortality for the babies of women who are victims of domestic violence [19]. Nogrum et al. [20] demonstrated that domestic violence was associated with prenatal and postnatal depression, complications of pregnancy, PTB and low birth weight. Despite the magnitude of the problem, few studies have been devoted to the investigation of the association between domestic and family violence against women and PTB.

Among the known causes of PTB are maternal infections. There is a consensus that infections trigger inflammatory responses in the mother and in the fetal tissues that may lead to the production of prostaglandins, increased myometral contractility, rupture of the fetal membranes, and consequent PTB [21–23]. However, no study has evaluated simultaneously the three infections most often implicated: bacterial vaginosis, periodontal disease, and urinary tract infection. Also, no studies have determined whether these three types of infection share the same profile of cytokine release, i.e., whether these cytokines act according to a determined physiopathological mechanism or whether there is a specific inflammatory response to each type of infection.

One of the unclear points in the epidemiology of PTB is the fact that some women experience an infectious stimulus, which, however, is unable to activate the inflammatory cascade and trigger PTB. It has been proposed that the inflammatory cascade may be activated only in genetically predisposed women [23]. Macones et al. [24] detected evidence of an association between genetic susceptibility in patients carrying polymorphism of the TNF-α gene (allele 2) and affected by symptomatic bacterial vaginosis with a higher risk of spontaneous premature delivery. However, the same was not observed in women with periodontal disease [25].

High rates of medical intervention in pregnancy also seem to contribute to an increased PTB rate. One in three of the more than 4 million births per year is by cesarean section and adjusted risk measurements may vary according to race and ethnicity [26]. Cesarean section rate has increased by more than 50% from 20.7% in 1996 to 32.8% in 2011 [27]. A parcel of medically induced deliveries appears to be indicated incorrectly, provoking iatrogenic prematurity [6, 7]; however, it is not known how much of the increase in PTB can be attributed to abusive intervention.

Although studies on aggravating factors are important, it is also necessary to better understand the protective factors. Social ties seem to influence the maintenance of health, favoring adaptive conducts in situations of stress [28, 29]. Studies have demonstrated that a lower social support during the prenatal period is associated with low birth weight and PTB [28, 30].

In the current protocol, the cited risk factors will be evaluated as components of three causality chains: the neuroendocrine, immunoinflammatory and medical intervention hypotheses.

**Figure 1:**
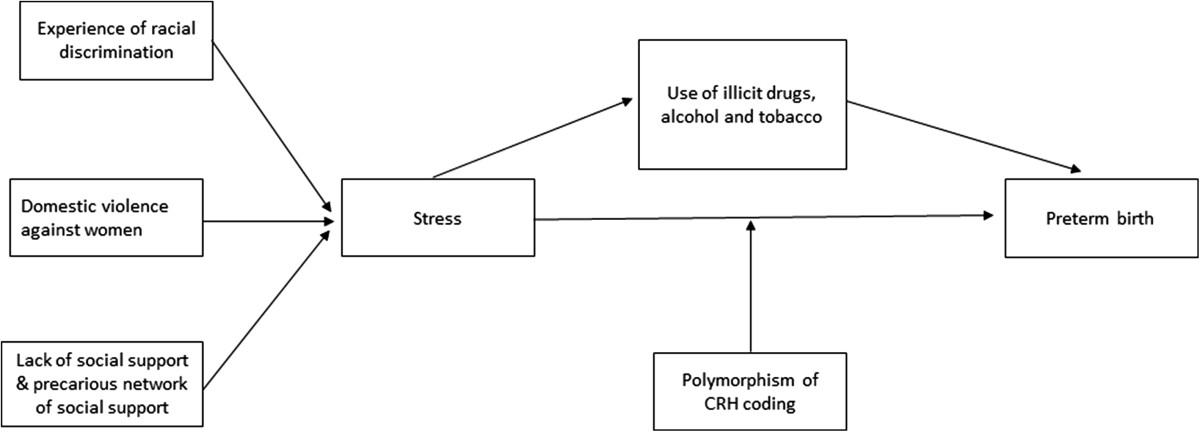
**Schematic diagram of the neuroendocrine hypothesis.**

The first hypothesis to be tested is the neuroendocrine one (Figure 1). Several psychosocial factors will be investigated: experience of racial discrimination, lack of social support, domestic violence against women, stress, and depression. The hypothesis is that all of these factors may act through a common physiological pathway, activating the hypothalamus-pituitary-adrenal axis and consequently the cascade of neuroendocrine events that promote stress, leading to the release of oxytocin and prostaglandin and inducing preterm delivery [31]. Another question to be investigated is whether genetically susceptible individuals with polymorphism in the region regulating corticotropin-releasing hormone (CRH) would be particularly predisposed to the occurrence of preterm delivery in the presence of stressful situations [11, 32].

**Figure 2:**
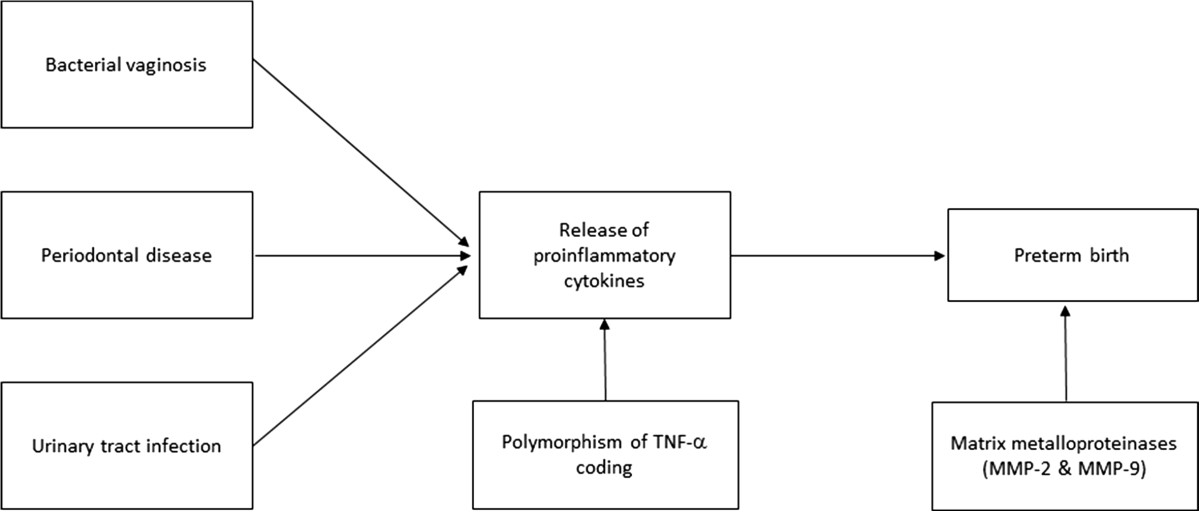
**Schematic diagram of the immunoinflammatory hypothesis.**

The second hypothesis is the immunoinflammatorry one (Figure 2). Three infections will be studied as possible stimuli capable of triggering the immunoinflammatory response that might lead to preterm delivery, i.e., bacterial vaginosis [33], periodontal disease [22, 34] and urinary tract infections [35]. Based on this hypothesis, the stimuli generated by these infections could lead to the release of proinflammatory cytokines (especially TNF-α and interleukins IL-1β, IL-6 and Il-10 in maternal blood), inducing an intra-amniotic inflammatory response that would trigger premature contractions, cervical dilation and rupture of fetal membranes, leading to preterm delivery [24, 25].

The third hypothesis to be tested is medical intervention. According to this hypothesis, there would be a reduction of stillbirths associated with an increase in low birth weight and PTB [6, 7, 36]. Women who undergo medical interventions would present higher PTB rates than all other pregnant women, adjusted for the remaining confounding factors [6, 5].

Thus, the objective of the present protocol is to investigate new factors in the etiology of PTB using an integrated and collaborative approach in two Brazilian cities in a cohort study started during the prenatal period. The following aspects will be evaluated as specific objectives considering the new hypotheses (neuroendocrine and immunoinflammatory): the association of domestic and family violence with PTB; to what extent the experience of racial discrimination promotes changes in the maternal organism that would trigger PTB; the influence of maternal stress during pregnancy on the birth of preterm babies; the association between maternal infections (bacterial vaginosis and urinary infection) and the triggering of PTB; the association of periodontal infection, presence of caries lesions and endodontic infections in the oral cavity of pregnant women with the occurrence of PTB; whether genetic susceptibility linked to polymorphism in the coding of TNF-α and CRH would confer a higher risk of preterm delivery.

## Methods

### Time period of the study

The protocol “Etiological factors of PTB and consequences of perinatal factors on the health of children: birth cohorts in two Brazilian cities”, denoted BRISA (Brazilian Ribeirão Preto and São Luís Birth Cohort Studies), conducted by the Federal University of Maranhão (UFMA) and the Faculty of Medicine of Ribeirão Preto, University of São Paulo (FMRP/USP), will be carried out in two Brazilian cities with contrasting socioeconomic indicators: São Luís and Ribeirão Preto.

The city of Ribeirão Preto (RP) is located in the Northeastern region of the State of São Paulo, a rich and industrialized region with a Human Development Index (HDI) of 0.800 in 2010, ranking in 40th position in Brazil [37]. Its population was 604,682 inhabitants in 2010 [38]. It is one of the most developed cities in the country, with 99% of all residences receiving piped water and equipped with a sewage system. Its main economic activity is sugar cane agro-industry, in addition to commerce and services. It is also a renowned regional university center [39].

The city of São Luís (SL), the capital of the state of Maranhão, is located in Northeastern Brazil. Its HDI was 0.768 in 2010, with the city ranking in 249^th^ position in Brazil. The city is located in one of the poorest regions in the country and its population was 1,014,837 inhabitants [38] in 2010. Its economic activity is based on aluminum metallurgy, ore export from the Carajás mountains and state production of soy, in addition to commerce and services [40].

### Protocol design and population

The reference population will consist of pregnant women receiving prenatal care at health services of the public and private networks. The goal is to obtain a convenience sample of approximately 1500 women in each city since it is not possible to obtain a random sample representative of pregnant women of the population because of the lack of registries of pregnant women or women receiving prenatal care.

A total of 1500 women will be invited for interview in each city during the prenatal period, from the 22^nd^ to the 25^th^ week of gestational age (GA). At the time of delivery, they will be reinterviewed after the birth of their babies. On that occasion, the hospitals of the two cities will be monitored daily for the identification of women belonging to the cohort.

Preterm birth is considered to be a birth occurring before 37 weeks of gestational age (GA). GA will be determined using an algorithm based on two criteria: date of last menstruation (DLM) and obstetric ultrasound (OUS) performed at less than 20 weeks of GA. When GA measured on the basis of DLM is 10 days more or less than the value estimated by OUS, the GA value calculated from DLM will be used or, otherwise, the GA estimated by OUS will be used [41].

This is a protocol for a prospective nested case–control study. The nested case–control design increases the efficiency of the study since there is no need to analyze biological specimens for all controls. All preterm babies identified in the cohort will be cases and two controls per case will be chosen at random from the rest of the cohort. This strategy reduces costs, practically maintaining the same statistical power equivalent to the analysis of the entire cohort. Only psychosocial variables and other variables obtained with a questionnaire during the interview (which require a prospective collection in order to reduce recall bias) will be measured for the entire cohort.

### Inclusion criteria

The inclusion criterion will be OUS performed before the 20^th^ week of pregnancy since it is a more reliable estimate of GA. The exclusion criterion will be multiple pregnancy, which has been pointed out as one of the main determinants of PTB, even in the absence of complications.

### Data collection and recruitment strategies

The women will be contacted in ultrasound clinics and prenatal care offices and invited to come to the Clinical Research Center of the University Hospital, Federal University of Maranhão, São Luís, or to the Clinical Research Unit of the University of São Paulo, Faculty of Medicine of Ribeirão Preto, to participate in the study. The project will be presented and disseminated in the media, and a telephone number will be provided for the interested mothers to contact the research team and schedule an interview according to the procedure used for a previous cohort [39].

All pregnant women who fulfill the inclusion criteria and who are interested in the project will be registered. The registration form contains identification data (name, address, personal telephone and telephones of family members), place were prenatal care visits were performed, and gestational age calculated by ultrasound.

After filling out the registration form, the subjects will receive a card with the date and time to arrive at the site of data collection, where the interviews will be held and the exams will be performed. At the site of data collection, the subjects will give written informed consent to participate in the study, respond to a general prenatal questionnaire and a self-applied questionnaire and will be submitted to anthropometry (weight, height), blood pressure measurement, collection of biological specimens (blood, urine and vaginal secretion), dental and ultrasound examination.

### Instruments and variables

Two standardized questionnaires will be used for data collection: one of them applied by the interviewers and the other self-applied.

The ABUSE–Assessment Screen questionnaire developed by the Nursing Research Consortium on Violence and Abuse [42] will be used as reference for the screening of physical and sexual abuse against pregnant women. A short version of the violence questionnaire of the World Health Organization will also be used. For psychological (emotional) violence, women will be asked whether since you were pregnant has someone: V1) insulted you or made you feel bad about yourself?; V2) belittled or humiliated you in front of others?; V3) intimidated or scared you on purpose?; V4) threatened to hurt you or somebody you care about? [19]. Regarding physical violence, the pregnant women will respond to the following questions: since you were pregnant has someone V5) slapped you or thrown something at you that could hurt you?; V6) pushed or shoved you, hit you with a fist or something else that could hurt?; V8) kicked, dragged or beaten you up?; V9) choked or burnt you on purpose?; V10) threatened you with, or actually used a gun, knife or other weapon against you? [19]. The last three questions deal with sexual violence: since you were pregnant V11) has someone ever physically forced you to have sexual intercourse against your will?; V12) have you ever had sexual intercourse because you were afraid of what your partner might do?; V13) has someone ever forced you to do something sexual you found degrading or humiliating? [19]. Violence will be identified when the pregnant woman respond “*onc* e”, “*seldom*” or “*often*” to one of the above questions.

Two instruments will be used to assess maternal stress: Perceived Stress Scale and reports of stressful life events. Stress will be considered to be present when >75^th^ percentile. The Center for Epidemiological Studies depression scale (CES-D) will be used to assess maternal depression. Severe depressive symptoms will be considered to be present when the score is ≥22. Intense anxiety will be identified when the pregnnat woman scores ≥30 points on the Beck anxiety Scale.

Experiences of racial discrimination will be evaluated by applying a questionnaire elaborated by Krieger [43] and used in the CARDIA [44] and the PRÓ-SAÚDE study in Brazil [45].

Social support and social support network will be evaluated using the social support scale of the Medical Outcomes Study (MOS). The scale was translated and adapted to Portuguese and later validated and used in the Pró-Saúde study on a cohort of employees of a Brazilian public University [46, 45]. Greater social support will be considered to be present when the replies are above the 75^th^ percentile.

The questionnaire, to be applied by the interviewer, contains questions about socioceconomic and demographic data (schooling, occupation, family income, marital status, religion, number of household members, and CCEB (Brazilian economic classification criterion in the Portuguese acronym), life habits (maternal use of alcohol, smoking habit and consumption of coffee, tea and chocolate), sexual and reproductive data (liveborns, stillborns and abortions), diseases and use of medications during pregnancy, characteristics of the current pregnancy and of prenatal care (receiving prenatal care; beginning of prenatal care; number of visits; place of prenatal care; presence of hypertension during pregnancy; chronic hypertension; chronic hypertension of the father and hypertension among family members; DLM; sexual and reproductive data (number of sex partners; sexually transmissible disease of the partner; use of contraceptive methods; type of contraceptive used); oral health data (oral health before pregnancy; gingival problems during pregnancy; changes in dental condition and indication of treatment by a dentist), use of fat-rich foods by means of the Block Score. The short version of the International Physical Activity Questionnaire (IPAQ) will be used to determine the physical activity of the pregnant women, classified as vigorous or heavy, moderate and light.

### Dental examination

The subjects will be submitted to clinical dental examination for the diagnosis of periodontal disease and caries. To this end, six examiners will be trained regarding the different research indexes (intraexaminer and interexaminer Kappa ≥0.80). All oral examinations will be performed in a dental office under artificial light according to WHO recommendations [47]. A no. 5 mouth mirror and a periodontal probe graduated in millimeters (North Carolina n°15/OMS #11.5, Hu-Friedy© 2013, Mfg. Co. LLC) will be used for the measurements.

Periodontal changes will be determined using the bleeding on probing (BOP) index [48], visible plaque index (VPI) [49], calculus index (CI) [50], probing pocket depth (PPD), and clinical attachment level (CAL) [51]. Because of the lack of a universally accepted criterion for the classification of periodontal disease [52], two different classifications will be used: a) the classification of the American Academy of Periodontology [53, 54] which considers three levels of severity of periodontal disease on the basis of clinical loss of attachment (CAL) as follows: slight =1–2 mm CAL; moderate =3 to 4 mm CAL; and severe =5 mm CAL; and b) another more specific criterion whereby a person is considered to have periodontitis when he/she presents at least two interproximal sites in different teeth with clinical attachment level ≥4 mm and/or at least two interproximal sites in different teeth, with probing depth ≥5 mm [55]. Dental caries will be determined only after dental prophylaxis and will be diagnosed on the basis of the DMFT index (number of decayed, missing and filled teeth) [56]. The presence of residual roots, fistulas or teeth with exposed crown chambers that might reflect endodontic infection will also be evaluated and diagnosed by clinical examination. The PUFA index (exposed Pulp, Ulceration of the oral mucosa by dislocated root fragments, Fistula and Abscesses) will be used to identify the presence of endodontic infection [57].

### Laboratory exams

Urine will be collected for the investigation of asymptomatic bacteriuria and urinary tract infection [58]. Asymptomatic bacteriuria will be identified by the presence of ≥100,000 colonies/ml in urine culture.

Vaginal secretion will be collected for the diagnosis of bacterial vaginosis and the diagnostic criterion is a Nugent score of 7 or more or the presence of clue cells [59–61].

The vaginal content will be collected and stored in Eppendorf tubes at −80°C for later processing and quantitation of metalloproteinases (MPPs) 2 and 9 and identification of Clamydia by PCR. After homogenization of the vaginal content, MMPs 2 and 9 will be determined by ELISA (Quantikine Elisa Kit – R&D Systems Human MPP-2 and ELISA Kit - Invitrogen– Human MMP-9). Clamydia will be identified by PCR.

The presence of *M. hominis and U. urealyticum*, as well as their antimicrobial susceptibilities, will be investigated with the commercially available Mycoplasma IST 2 Kit (bioMérieux, Marcy-l’Étoile, France) as indicated by the manufacturer. Briefly, the endocervical cotton swab included in the kit will be inoculated in R1 transport medium, inhibiting most of the Gram-negative and Gram-positive bacteria. The inoculated R1 medium will be vortexed rapidly and 3 mL will be added to the growth R2 medium, which contains 1 mL of lyophilized urea/arginine broth. After reconstitution and shaking, 55 μL will be dispensed into each of the 22 test wells on the strip. Two drops of mineral oil will be added to each well. The remainder of the R2 medium and the inoculated strip will then be incubated at 37°C and observed for color changes at 24 and 48 h. The antimicrobial susceptibility testing will include tetracycline, doxycycline, erythromycin, azithromycin, clarithromycin, josamycin, ofloxacin, ciprofloxacin and pristinamycin. The development or absence of red colour on the relevant part of the strip provides an index of resistance or susceptibility to each antimicrobial agent, respectively, according to the guidelines of the CLSI.

Thirty-two ml blood will be collected by peripheral venipuncture into four tubes containing EDTA (purple), four yellow tubes containing a gel activator, and one blue tube and sent to the laboratory. Blood will be centrifuged for the separation of serum, plasma and whole blood. The material obtained will be placed in Eppendorf tubes labeled and stored frozen at −80°C. The whole blood fraction will be used for DNA extraction for the study of TNF-α and CRH gene polymorphism. The serum fraction will be used to quantitate the levels of 42 pro- and anti-inflammatory cytokines with a high sensitivity kit (Milliplex Map Human Cytokine/Chemokine. Cat HCYTOMAG-60K-PX41 Millipore Corporation, Billerica, MA, USA) and TGF-β will be determined by ELISA (Elisa Human TGF Beta-1 R&D System). Plasma will be used to quantitate MPPs 2 and 9 by ELISA (Quantikine Elisa Kit – R&D Systems Human MMP-2 and ELISA Kit - Invitrogen– Human MMP-9).

### Detection of TNF-α and CRH gene polymorphism

For the analysis of TNF-α and CRH gene polymorphism, genomic DNA will be extracted from peripheral blood prenatally obtained from the pregnant women according to the adapted technique of Sambrook, Fritsch and Maniatis [62]. The alleles at position – 307 of the promoter region of gene TNF-α will be detected by PCR-RFLP. A 107 bp fragment will be amplified using the primers 5`AGGCAATAGGTTTTGAGGGCCAT-3` and 5`TCCTCCCTGCTCCGATTCCG-3` (GDB:196368). The total volume of the amplification reaction will be 25 μL, containing 1 μL genomic DNA (25 ng), 1X buffer (Invitrogen), 0.25 pmol of each primer, 0.05 μM dNTP, 1.5 mM MgCl_2_ and 1 U Taq DNA polymerase. PCR will be started with 5 minutes of denaturation at 95°C, followed by 35 cycles of 20 seconds at 95°C, 20 seconds at 59°C and 20 seconds at 72°C. PCR will be finalized by a final extension for 10 minutes at 72°C.

Digestion of the 307G allele by the enzyme *Nco* I produces fragments of 87 and 20 pb, while the 307A allele is not cleaved by the enzyme, generating a fragment of 107 bp [63, 24]. The amplified fragments will be digested for 3 hours at 37°C, submitted to 3% agarose gel electrophoresis and visualized by ethidium bromide staining.

Polymorphism of the 5` flanking region of the CRH gene will be determined by digestion with the enzyme *Xmn* I, which permits the detection of the variant (T255G) [64].

### Obstetric ultrasonography

On the day of collection of clinical and laboratory data, all pregnant women will be submitted to morphological OUS with analysis of fetal biometry and morphology and uterine artery Doppler using a transducer by the abdominal route. They will also be evaluated in terms of the length and biophysical characteristics of the uterine cervix using a transducer by the vaginal route.

### Statistical analyses

The primary outcome will be preterm birth (<37 weeks of GA). Data will be analyzed by multiple binary logistic regression with estimate of the odds ratio. Modeling with structural equations will also be used to reduce measument errors. This form of hierarchical modeling has been used to interpret complex relationships between variables and has advantages over classical regression analysis. It is more appropriate by permitting an estimate of direct and indirect effects (mediation effect) and a better control of confounding factors by also adjusting for common causes. When the variables are measured in an imperfect manner (as is the case for most psychosocial variables such as stress, experiences of racial discrimination and social support), latent variables are created. These latente variables, which are not directly observed, are derived from the structure of covariance between two or more indicator variables (observed variables are used to estimate the latent variable). The latent variables are free of measurement errors. With the use of this modeling it is possible to construct a network of structural relations arranged in a hierarchical manner into distal, intermediate and proximal causes based on the hypothesis derived from theory, and to test the adjustment of the model as a whole. Modeling with structural equations consists of a series of multiple regression equations, with all equations being adjusted simultaneously. Various scales used in the study will be validated by confirmatory factor analysis before being used in the complete structural equation model [65].

### Sample design and statistical power

The reported prevalences of the explanatory variables range from 10 to 50%. Thus, considering a 12% PTB rate, the initial plan is to recruit 3000 women (1500 in ecah city), a number that would lead to a total of 360 cases. The nested case control will thus be based on 360 cases and 720 controls (two controls per case), with a 5% probability of type I error and an 80% power to detect an odds ratio of 1.7 associated with a prevalence as low as 12% of the explanatory variables, even in the presence of moderate confounding (OR of 1.8 for confounding).

### Data processing

Data will be entered in duplicate using the Microsoft Office Access 2007 software. The two sets of entries will be compared and errors corrected. Inconsistencies will be corrected.

### Ethical considerations

The protocol satisfies the criteria of Resolution 196/96 of the National Health Council and its complementary norms and was approved in all of its phases by the Research Ethics Committe of the University Hospital, FMRP/USP (Protocol N°. 4116/2008) and of the University Hospital of the Federal University of Maranhão (Protocol N°. 4771/2008-30). The mothers who agree to participate in the study will give written informed consent and will be informed that they could drop out at any time during the study with no harm to themselves or their families.

## Discussion

The proposed protocol focuses on the question of PTB, whose rates have continued to be increasing in both developed and developing countries for more than a decade [1, 2]. No current intervention has had an appreciable impact on the reduction of this event [66–70]. Thus, there is a need to explore new hypotheses and mechanisms of causality using an integrated approach, collaboration among research groups, and less fragmented theoretical-methodological approaches.

New risk factors for PTB, such as experience of racial discrimination, stress during pregnancy, domestic violence against women, lack of social support, maternal infections (bacterial vaginosis, infections of the oral cavity and urinary tract infections) genetic susceptibility (polymorphisms in the coding of TNF-α and CRH), and medical interventions will be evaluated under the present protocol [12, 13, 17, 20, 25, 33].

The classical risk factors for PTB only explain part of its occurrence [8]. New hypotheses need to be tested so that effective interventions are proposed. Psychosocial, genetic and infectious mechanisms were selected for investigation since there are indications that they play an expressive role in the determination of PTB.

The proposals of the study at the two locations will be articulated with each other, both in terms of the research teams of the two centers and the use of the same instruments. The study will be conducted in two Brazilian cities with discrepant socioeconomic realities.

The expectation is to identify risk factors for PTB having a greater predictive power than classically studied factors. After the identification of these factors, the intention is to design more specific intervention strategies to be tested. In the current literature, risk factors have been tested individually, with few protocols having been proposed like the present one, in which the factors are investigated using an integrated multidisciplinary approach and a hierarchical modeling based on data from samples of two cities with contrasting socioeconomic profiles. The final objective is to propose more effective strategies for the reduction of PTB, which, after being tested, may be useful for the elaboration of health policies.

## Authors’ original submitted files for images

Below are the links to the authors’ original submitted files for images. Authors’ original file for figure 1 Authors’ original file for figure 2
