# Supplementary figures and images for: A protocol to identify non-classical risk factors for preterm births: the Brazilian Ribeirão Preto and São Luís prenatal cohort (BRISA)
Source: Reprod Health. 2014 Nov 19;11:79. doi: 10.1186/1742-4755-11-79 (PMC4246428; doi:10.1186/1742-4755-11-79)

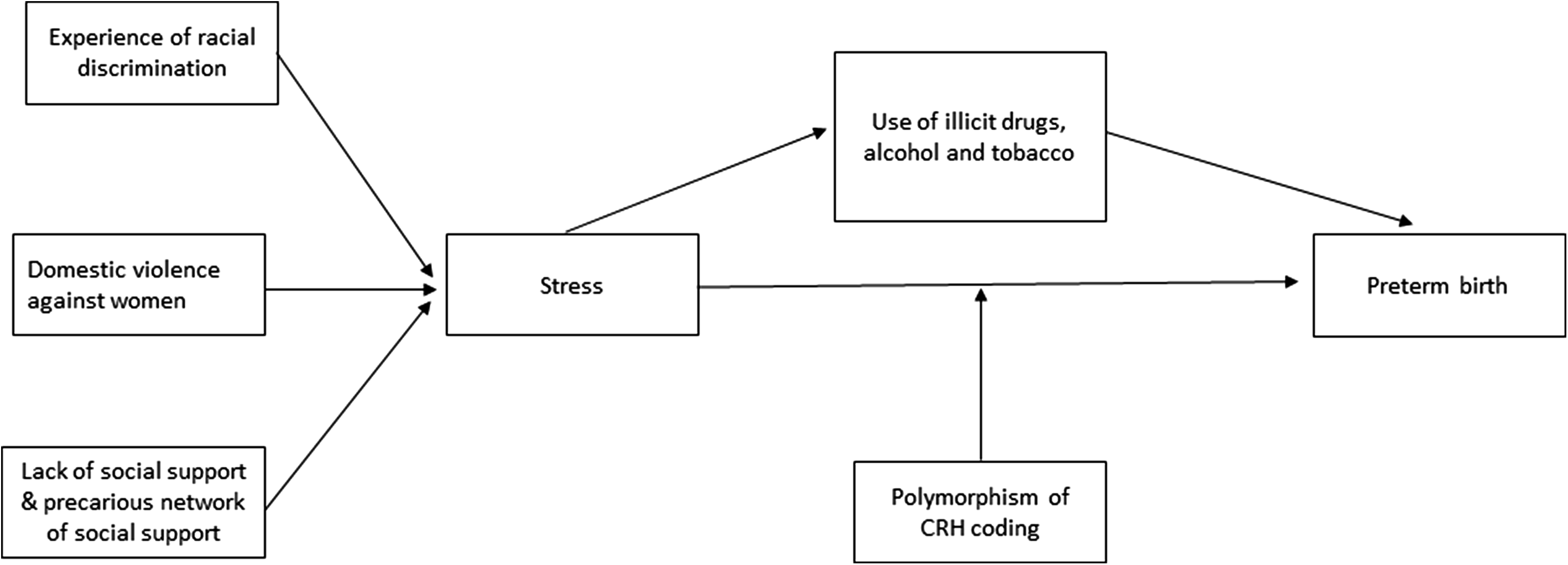

Supplement: Supplementary file 1 — Authors’ original file for figure 1 [file 12978_2014_328_MOESM1_ESM.tif]

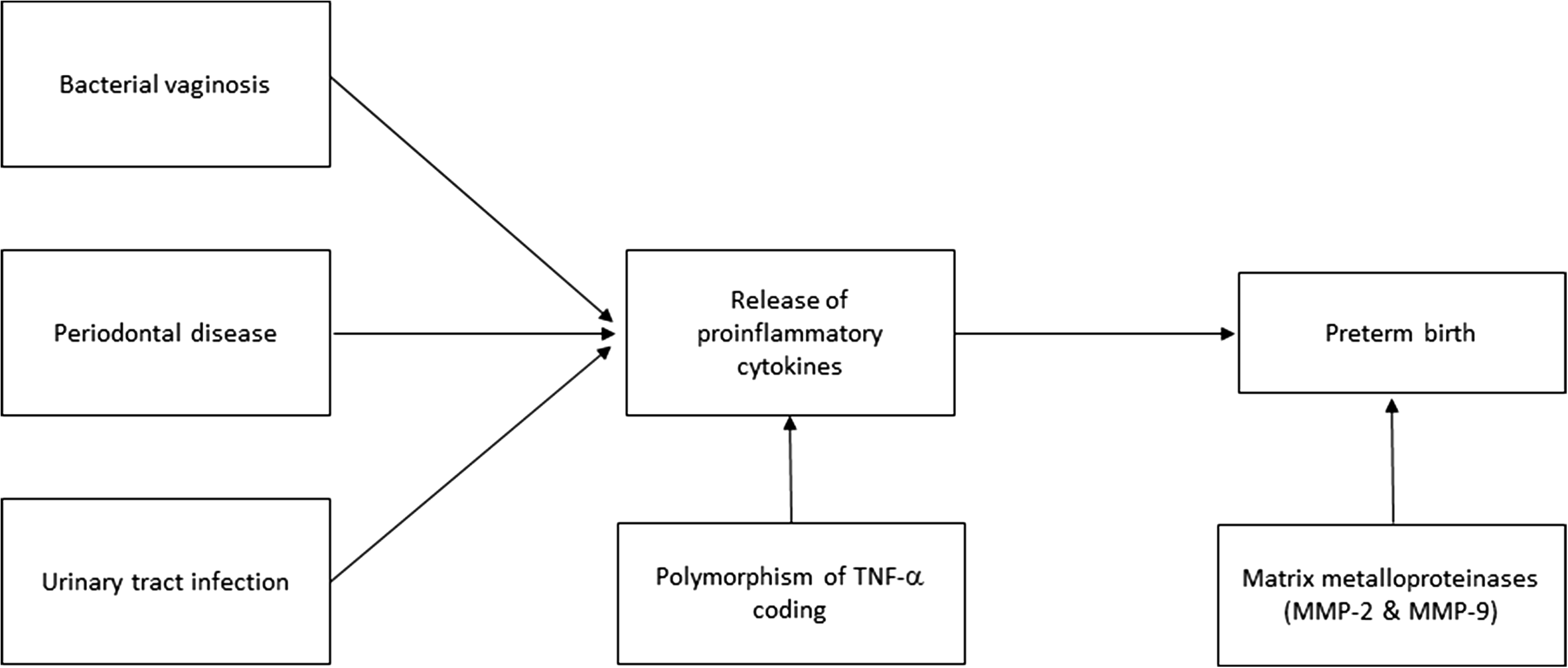

Supplement: Supplementary file 2 — Authors’ original file for figure 2 [file 12978_2014_328_MOESM2_ESM.tif]
